# A Fluorescent Glycolipid-Binding Peptide Probe Traces Cholesterol Dependent Microdomain-Derived Trafficking Pathways

**DOI:** 10.1371/journal.pone.0002933

**Published:** 2008-08-13

**Authors:** Steffen Steinert, Esther Lee, Guillaume Tresset, Dawei Zhang, Ralf Hortsch, Richard Wetzel, Sarita Hebbar, Jeyapriya Raja Sundram, Sashi Kesavapany, Elke Boschke, Rachel Kraut

**Affiliations:** 1 Institute of Bioengineering and Nanotechnology, A*Star, Singapore, Singapore; 2 Department of Biochemistry, Neurobiology Programme, National University of Singapore, Singapore, Singapore; 3 Institut für Lebensmittel- und Bioverfahrenstechnik, Technische Universitaet Dresden, Dresden, Germany; University of Geveva, Switzerland

## Abstract

**Background:**

The uptake and intracellular trafficking of sphingolipids, which self-associate into plasma membrane microdomains, is associated with many pathological conditions, including viral and toxin infection, lipid storage disease, and neurodegenerative disease. However, the means available to label the trafficking pathways of sphingolipids in live cells are extremely limited. In order to address this problem, we have developed an exogenous, non-toxic probe consisting of a 25-amino acid sphingolipid binding domain, the SBD, derived from the amyloid peptide Aβ, and conjugated by a neutral linker with an organic fluorophore. The current work presents the characterization of the sphingolipid binding and live cell trafficking of this novel probe, the SBD peptide. SBD was the name given to a motif originally recognized by Fantini et al [1] in a number of glycolipid-associated proteins, and was proposed to interact with sphingolipids in membrane microdomains.

**Methodology/Principal Findings:**

In accordance with Fantini's model, optimal SBD binding to membranes depends on the presence of sphingolipids and cholesterol. In synthetic membrane binding assays, SBD interacts preferentially with raft-like lipid mixtures containing sphingomyelin, cholesterol, and complex gangliosides in a pH-dependent manner, but is less glycolipid-specific than Cholera toxin B (CtxB). Using quantitative time-course colocalization in live cells, we show that the uptake and intracellular trafficking route of SBD is unlike that of either the non-raft marker Transferrin or the raft markers CtxB and Flotillin2-GFP. However, SBD traverses an endolysosomal route that partially intersects with raft-associated pathways, with a major portion being diverted at a late time point to rab11-positive recycling endosomes. Trafficking of SBD to acidified compartments is strongly disrupted by cholesterol perturbations, consistent with the regulation of sphingolipid trafficking by cholesterol.

**Conclusions/Significance:**

The current work presents the characterization and trafficking behavior of a novel sphingolipid-binding fluorescent probe, the SBD peptide. We show that SBD binding to membranes is dependent on the presence of cholesterol, sphingomyelin, and complex glycolipids. In addition, SBD targeting through the endolysosomal pathway in neurons is highly sensitive to cholesterol perturbations, making it a potentially useful tool for the analysis of sphingolipid trafficking in disease models that involve changes in cholesterol metabolism and storage.

## Introduction

Sphingolipids segregate into nano-scaled domains at the plasma membrane, commonly referred to as lipid rafts, which are defined by high sphingolipid and cholesterol content, and low buoyant density in high-speed ultracentrifugation gradients [2]–[5]. Rafts are now thought to include a variety of plasma membrane domains with different characteristics that invaginate into endocytic vesicles [6]–[11].

A method that would allow the tracking of intracellular pathways taken by raft-borne sphingolipids is of general interest, because of their involvement in a number of biologically and clinically important processes. Sphingolipid and cholesterol trafficking is altered in the cells of patients with Niemann Pick disease, and a number of other lipid storage diseases where sphingolipids accumulate in late endosomal and lysosomal compartments [12]–[14]. Cholesterol and sphingolipids such as ceramide, sphingomyelin, and gangliosides are also thought to be involved in the pathogenesis of Alzheimer's disease [15]–[18]. Many viruses and pathogens, including the Alzheimer's associated amyloid peptide, recognize specific carbohydrate-containing headgroups of glycosphingolipids [19]–[22], a large variety of which are expressed on the surfaces of cells and occupy membrane microdomains [23]–[27]. Therefore, a sphingolipid-targeted, exogenous probe for live imaging studies would be a useful tool in studying diseases whose pathogenesis is glycosphingolipid-dependent. Currently, non-invasive small-molecule tracers that can be used to visualize the binding and trafficking of sphingolipid-containing microdomains are not available.

The most commonly used sphingolipid-binding probe, Cholera toxin B (CtxB), may be atypical in that it can be internalized by both non-clathrin and clathrin-dependent uptake mechanisms [28]–[30]. It is also important to consider that CtxB induces clustering of sphingolipids [31]–[33], and binds very tightly and specifically to a single target glycolipid, GM1 [34]. CtxB and another common microdomain tracer, the glycosyl-phosphatidyl-inositol (GPI)-anchor fused to fluorescent protein, both traffic primarily to the Golgi [35] (although this has been contested [36]), and may occupy primarily non-raft domains [37], [38]. Other tracers of non-clathrin uptake pathways currently in use are green fluorescent protein (GFP) fusions of the endocytic adaptors Flotillin and caveolin [39]–[41]. Fluorescent protein fusions have the disadvantage that they must be expressed from transgenes, and therefore may fluoresce in the biosynthetic pathway. Additionally, these endocytic adaptor proteins are not found universally in all cell types, and correspond to a specific subtype of membrane microdomain [11].

With this background, our goal was to generate a probe with which one could monitor changes in raft-derived sphingolipid trafficking that occur in lipid-related and neurodegenerative disease states. To this end, we created a small fluorescently coupled peptide probe consisting of a domain, the sphingolipid binding domain, SBD, which Fantini and coworkers identified in the sequences of several proteins that were known to bind to cell-surface glycosphingolipids [22]. The Alzheimer's disease-associated Aβ peptide contains a variant of the V3 loop domain, which is also found in HIV-1 gp120 and the Prion protein (Prp) (ibid). This domain can bind to synthetic membranes containing sphingolipids [22], [42]–[45] and associations have been reported between V3 loop-bearing proteins and gangliosides 21,22,46–50. In a previous study [51] we reported that a myc-tagged version of the 25 amino acid SBD peptide derived from Aβ [22] is associated with detergent-resistant membranes in neurons, and showed that cholesterol depletion inhibits uptake and alters the diffusion behavior of fluorescently tagged SBD at the plasma membrane, which is normally slow. In that study, we also demonstrated a dependence on sphingolipids for endocytosis, and an interaction with immobilized sphingolipids in fat blots.

Here, we present evidence in support of the idea that fluorescent SBD acts as a sphingolipid tracer. Fluorophore-coupled SBD binds preferentially to artificial membranes that contain a raft-like mixture of sphingomyelin, cholesterol, and gangliosides. However, SBD differs from CtxB in its ganglioside binding properties in that it does not display a strong preference for a particular ganglioside. Time-course quantitative colocalization of the SBD probe with a variety of live fluorescent markers shows that SBD is rapidly incorporated into early endosomes of mammalian and Drosophila neurons. Similarly to fluorescent lactosyl-ceramide and other sphingolipid-associated markers such as caveolin-GFP and CtxB [52], [53], SBD's trafficking route is strongly altered by cholesterol perturbations, but unlike these markers, it appears to cycle between the endolysosomal and recycling pathways.

The fact that SBD labels Drosophila as well as mammalian neurons effectively make it a useful tool for analyzing sphingolipid trafficking pathways in disease models in this genetically tractable organism. Although SBD is derived from the amyloid peptide Aβ, it is non-toxic, and can therefore be used to trace sphingolipid trafficking pathways in a non-invasive way.

## Results

### I. SBD is recognized specifically and taken up by neuronal cells in culture

**Figure 1:**
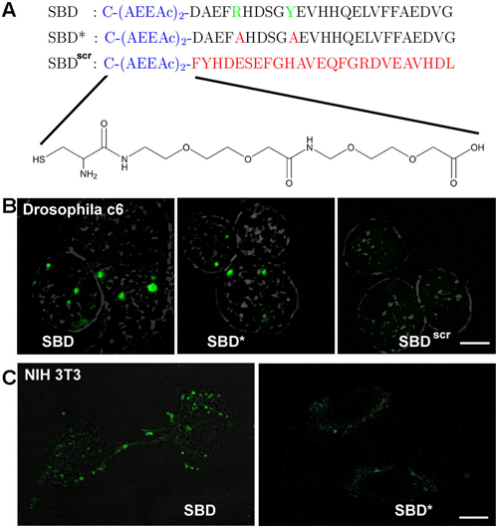
SBD binds to and is internalized by insect and mammalian cells. A. Sequences of the SBD, SBD*, and SBD^scr^ peptides with amino-terminal Cysteine and AEEAc spacer. For SBD-TMR, the fluorophore was conjugated directly via amine linkage to the spacer. B. Drosophila c6 neuronal cells labelled with SBD-, SBD*-, or SBD^scr^-Oregon Green (conjugated with SBD at the terminal Cysteine) at 10 uM in HBSS for 15 min at 25°C. Wild type SBD shows internalized punctae representative of endocytic domains, whereas the number and intensity of punctae in SBD* and SBD^scr^ are much reduced. C. Mouse NIH3T3 fibroblasts labelled with SBD-OG or SBD*-OG at 2 µM in HBSS for 15 min at 37°C. Scalebar in B = 5 µm; in C = 10 µm.

A sphingolipid binding domain (SBD) peptide of 25aa was generated based on a motif identified by Mahfoud et al, which occurs in several viral and pathogenic proteins that co-purify with glycosphingolipids [22], [42]. The motif contained within the Aβ peptide was modified by a neutral diethylene glycol linker to facilitate conjugation of fluorophores to the amino-terminus and to minimize steric interference of the fluorophore with binding of the peptide. SBD (fig. 1A; see Methods) was coupled to the small molecule fluorophores Oregon Green (OG) and tetramethylrhodamine (TMR) for live cell imaging.

Since the SBD marker has potential applications as a sphingolipid trafficking tracer for cellular and animal models of neurodegenerative disease, we examined the distribution of the marker in several different cell types, including insect and mammalian neurons.

SBD-OG or SBD-TMR, which was shown to be non-toxic at and above the range of concentrations used for labelling experiments (see fig. S1 and Methods), was applied to adherent mammalian NIH-3T3 fibroblasts, neuroblastoma SH-SY5Y [54], and primary mouse cortical neurons [55]. Uptake after incubation at physiological temperature was recorded by confocal and wide-field fluorescence imaging in time-lapse. SBD is taken up rapidly (<5 minutes) into vesicles that are similar in size and distribution (fig. 1B, C) to endosomes labeled by Dextran and lysotracker (see movie in neuroblastomas, fig. S4). SBD uptake and trafficking was also analyzed in Drosophila neurons, because we are interested in using it as a sphingolipid/microdomain-directed probe for analysis of genetic disease models in the fly that affect sphingolipid trafficking and storage. In Drosophila DL-DmBG2-c6 neurons (hereafter called c6) [56], in contrast to mammalian cells, little SBD fluorescence is seen at the plasma membrane, although SBD is taken up readily by these neurons at physiological temperature (25°C). At 4°C the labeling is inefficient, indicating that the interaction of SBD with the plasma membrane is inhibited by low temperature (not shown). SBD appears to have a higher affinity for mammalian cells, since only about one-fifth of the concentration was required for labelling as for Drosophila cells (fig. 1B, C; see Methods). This is not surprising, since Drosophila do not produce gangliosides [57], which are the presumed optimal receptors for SBD, but they do have other glycolipids.

As a control for the specificity of the SBD interaction with the plasma membrane, we also tested two mutated forms of the peptide (fig. 1A): SBD* contains mutations in two amino acids (R_5_→A and Y_10_→A) that were postulated by Fantini and colleagues to mediate electrostatic and π-bonding interactions with glycosphingolipids [22], [42]; SBD^scr^ (scrambled) contains the same amino acids as SBD in a random sequence. SBD* is taken up less than half as efficiently as SBD, judging by the appearance of <50% fewer punctae and lower fluorescence intensity within cells (fig. 1B, C; quantitation not shown), whereas SBD^scr^ does not label cells at all unless added at concentrations of 50 µM or more (fig 1B). SBD-TMR and SBD-OG colocalize nearly completely (∼80%; data not shown), demonstrating that the fluorophore does not influence the localization of SBD. SBD-TMR, SBD-OG, and non-fluorophore tagged SBD also behaved similarly with respect to their membrane-association properties in biophysical assays (see section II, and Hebbar et al [51]).

The integrity of the SBD peptide after uptake was measured by dot-blotting cell extracts of SH-SY5Y after uptake of a myc-tagged version of the probe, and incubation with anti-myc (fig. S2). This showed that 90% and 85% of SBD-myc were detectable after 30 min and 60 min post-5 µM incubation and chase, respectively. This was comparable to the 87% and 86% of CtxB-peroxidase that was detectable after 1 µg/ml incubation and 30 min or 60 min chase.

### II. SBD binds to gangliosides in liposomes with raft-like composition

Previously, we assessed SBD binding to various lipids by lipid-protein overlay (fat-blot) assay, and found no detectable interaction with glycosylated and non-glycosylated sphingoid bases, sphingosylphosphocholine, ceramide, sulfatide, cholesterol, glycerophospholipid, and myriocin [51]. In fat blots, SBD bound gangliosides GM1, GD1a, GD1b, and slightly with SM [51]. However, we wished to assess binding to combinations of sphingolipids and gangliosides in a more membrane-like setting that would support microdomain formation.

**Figure 2:**
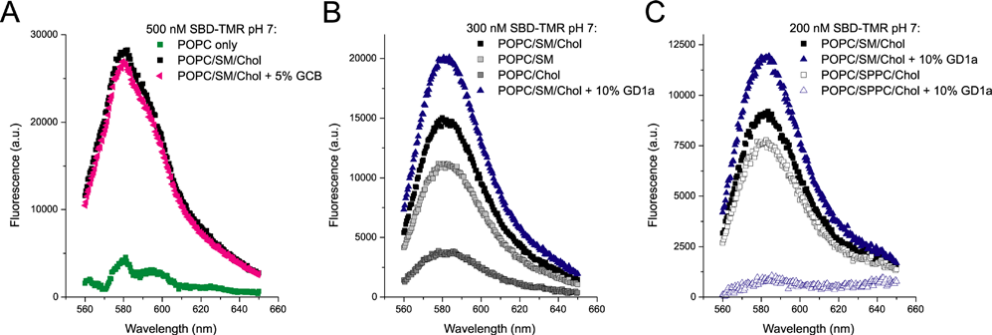
SBD binds preferentially to glycolipid-containing liposomes with raft-like composition. A. Fluorescence retained on liposomes of different composition after binding SBD-TMR to liposomes and filter-separation of unbound probe. SBD-TMR was retained much more strongly on liposomes containing the raft-like mixture of POPC∶SM∶Ch (45∶25∶30) (black squares) than on liposomes composed only of POPC (green boxes). Addition of 5% GCB (pink triangles), did not improve binding. B. A POPC/Chol (45∶55) mixture (dark gray boxes) showed the lowest binding capacity, whereas a POPC/SM (45∶55) mixture bound with intermediate affinity (light gray boxes). This was further improved by the presence of cholesterol (black boxes) and 10% GD1a (blue triangles). C. SBD shows some binding to the saturated glycerophospholipid SPPC, when substituted for SM in the basic raft mixture (POPC∶SPPC∶Ch 45∶25∶30) (white boxes). Unlike SM containing rafts, however, binding is abolished by addition of 10% GD1a (blue empty triangles). In all graphs, background fluorescence in the absence of liposomes, (e.g. due to possible retention of SBD in aggregated form) was subtracted. In POPC/SM/Chol liposomes, background fluorescence accounted for ∼25% of total signal.

To achieve this, we employed unilammelar liposomes of ∼100nm average diameter, consisting of a raft-like base mixture of POPC/SM/Chol (Palmitoyl-Oleoyl-Phosphatidyl-Choline/Sphingomyelin/Cholesterol 45∶25∶30 mol%) [58], with various components substituted, added, or subtracted. First, the requirement for SM and cholesterol was tested, with or without galactosyl-cerebroside, which had been suggested to bind SBD domains in general by Fantini et al [22]. After incubation with SBD-TMR for 15 min at 37°C and filtration of the bound liposomes, fluorescence remaining associated with the liposomes was quantified by spectrofluorimetry, and the background level (without liposomes) was subtracted. After background correction, about six times more SBD-TMR was associated with the SM- and cholesterol-containing liposomes than the POPC-only liposomes, but this was not enhanced by the presence of 5% galactosyl-cerebrosides (a mixture of single-galactose sphingolipids) (fig. 2A). This is in contrast to the fat-blot assays [51], where galactosyl-cerebrosides do bind strongly to SBD. We next left either cholesterol or SM out of the mixture, showing that SBD required both SM and cholesterol together for the best binding, although SM alone provided some affinity (fig. 2B), in agreement with fat blots [51]. Addition of 10% GD1a to the basic raft mixture enhanced the binding still further (blue trace in fig. 2B).

To test whether SBD could bind to domains consisting of non-sphingolipid saturated lipids, we combined the double-saturated glycerophospholipid SPPC (Stearyl-Palmitoyl-Phosphatidyl-Choline) with cholesterol in a POPC background, with or without added 10% GD1a (fig. 2C). While the binding to POPC/SPPC/Chol was slightly less avid than to the standard POPC/SM/Chol mixture, addition of the glycosphingolipid drastically inhibited binding, indicating that the interaction of SBD with glycosphingolipids is highly dependent on the simultaneous presence of SM.

**Figure 3:**
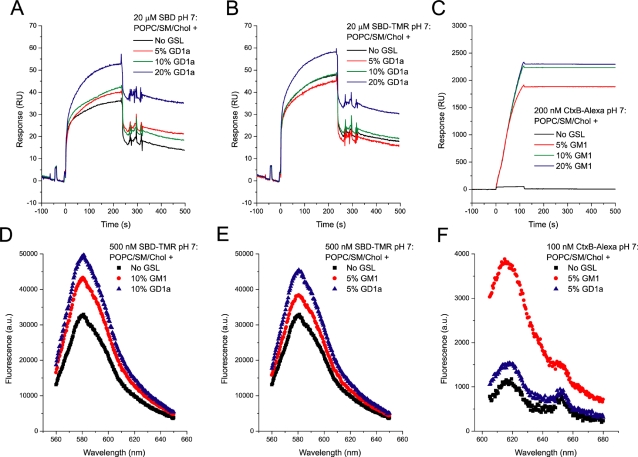
SPR (A–C) and spectrofluorimetric (D–F) binding assays of SBD-TMR to a POPC/SM/Chol (45∶25∶30 mol%) mixture show that a high concentration of ganglioside (10–20%) is required for optimal binding, by comparison with CtxB to its target, GM1 (C, F). A, B. Both non-fluorescently coupled SBD (A) and SBD-TMR (20 µM) (B) bind more strongly to 20% GD1a-containing POPC/SM/Chol liposomes immobilized on a Dextran-coated L1 sensorchip. C. CtxB-Alexa488, in contrast to SBD, binds at a lower concentration (200 nM) with significantly higher affinity. After the injection, CtxB showed nearly no dissociation from the liposome substrate, as indicated by a continued high response level. D, E. Comparison between SBD-TMR (500 nM) binding to POPC/SM/Chol liposomes containing no ganglioside (black ▪) vs. GM1 (red •) or GD1a (blue ▴), at 10% or 5% (D, E respectively) by spectrofluorimetric assay. F. Similar liposome assay as in D, E, with 100 nM of CtxB-Alexa binding to POPC/SM/Chol liposomes with no ganglioside (black ▪) vs. 5% GD1a (blue ▴) or 5% of GM1 (red •). For spectrofluorimetric curves (D–F), the background fluorescence in the absence of liposomes was substracted.

Next, SBD's preference for different concentrations of two glycosphingolipids that had been reported to interact well with Aβ, GM1 and GD1a [46], [48], [59], was tested, and compared with the binding of CtxB to the same two glycosphingolipids (fig. 3). Marginally more SBD-TMR remained bound to liposomes that contained a higher content of glycosphingolipid, up to 10% in the spectrofluorimetric assay, and GD1a was slightly preferred over GM1 (fig. 3D, E). SBD binding to gangliosides was also tested by surface plasmon resonance (SPR) assay, using the Biacore 3000 and L1 Dextran-coated gold sensorchips (GE Healthcare) on which the POPC/SM/Chol liposomes were immobilized. SPR demonstrated much improved binding to 20% GD1a (fig. 3A, B). This is in contrast to the binding behavior of CtxB-Alexa594, which bound hardly better to GD1a than to no glycosphingolipid, bound nearly maximally to only 5% GM1, and unlike SBD did not detach (fig. 3C). By SPR, uncoupled SBD (fig. 3A) and SBD-TMR (fig. 3B) gave very similar binding curves, suggesting that the presence of the fluorophore on SBD does not drastically change its binding properties. The binding affinity of SBD to 10% GD1a-containing POPC/SM/Chol liposomes at pH 7 was calculated by SPR to be between 2×10^−7^ and 4×10^−6^ (K_D_ calculated from fitting curves at various concentrations are shown in fig. S3). This is similar to what has been reported for Aβ1-40 [49].

**Figure 4:**
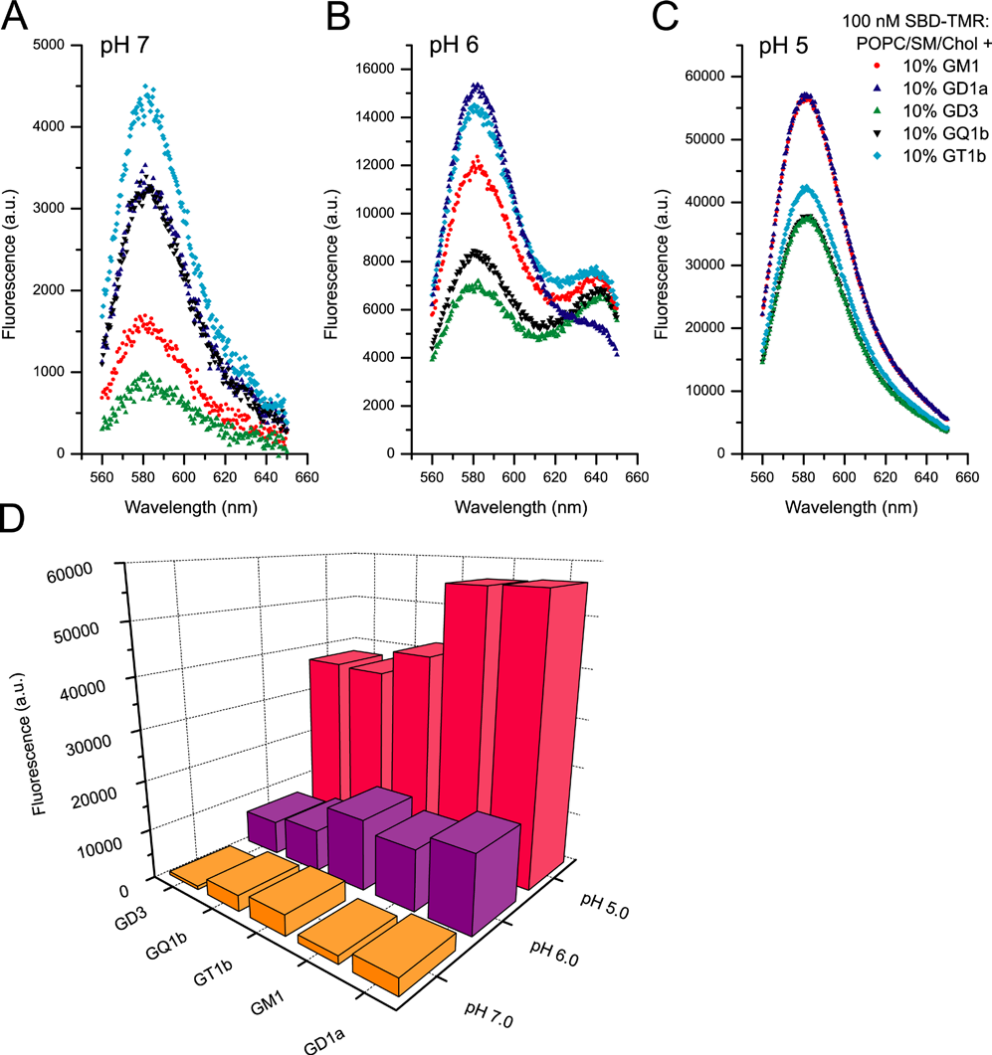
SBD preference for particular gangliosides depends on pH, and interaction with raft-ganglioside mixtures is strongly enhanced at low pH. Spectrofluorimetric liposome binding assay of 100 nM of SBD-TMR with the raft-like ternary mixture POPC/SM/Chol (45∶25∶30 mol%). 10% of the given ganglioside was incorporated into the lipid membrane: GM1 (red •), GD1a (blue ▴), GD3 (green ▴), GQ1b (black ▾) and GT1b (cyan ♦). A, B and C are the fluorescence spectra taken at pH 7, 6 and 5 respectively. D summarizes the comparative fluorescence responses at 580 nm for the various gangliosides at different pH.

Previous reports using SPR had found that binding of Aβ(1–40) to gangliosides increased with sialylation, i.e. that more highly sialylated forms such as the GQ or GT series bound more tightly [47]. In order to compare SBD's binding preference for different gangliosides, and the effect of pH on binding efficiency, liposomes were made with the standard POPC/SM/Chol (45∶25∶30 mol%) mixture plus 10% of GM1, GD1a, GD3, GT1b, or GQ1b, and incubated with SBD at pH 5, 6, or 7 (fig. 4A–C). At neutral pH, SBD bound better to the triply sialylated GT1b in comparison to the less sialylated forms, GD3 (disialylated) and GM1 (monosialylated). At lower pH (with an optimum at pH 5), binding to all glycosphingolipids was strikingly improved (see summary 3D plot in fig. 4D), and interestingly, interactions with less sialylated gangliosides GM1 and GD1a became stronger.

Binding of SBD was also examined in the glycolipid-deficient melanoma cell line GM95 [60], but was taken up very inefficiently by both this cell line and its wild type parental line, B16 (not shown). However, pharmacological inhibition of glycolipid synthesis in SH-SY5Y using Fumonisin B1, which inhibits ceramide synthase, did result in reduced binding and uptake of SBD in neuroblastomas (described in [51]), indicating that sphingolipids are necessary for uptake. The low level of labeling seen in B16 and GM95 melanoma may represent the amount of uptake that is mediated by the relatively weak interaction of SBD with POPC, SM and cholesterol alone (see fig. 2, 3). Reports that B16 melanomas produce almost entirely GM3 (∼95%), with the remainder consisting of GM2, GM1, and possibly GD1a [61] support the conclusion that SBD indeed prefers high concentrations of more complex ganglioside species (such as GT1b, GD1b, or GQ1b; see fig. 4) in which this cell line is deficient.

### III. The SBD uptake pathway is distinct from that of known microdomain markers and Transferrin

In order to characterize SBD's trafficking route, we carried out time-course quantifications of SBD's colocalization after pulse chase labeling at physiological temperature, with respect to reference microdomain-localized and endolysosomal markers. Quantification was done using the colocalization algorithm of Costes [62], to obtain the percentage intensity, from the Manders coefficient (tM) [63] of the SBD signal over a given threshold that is also positive for the reference marker (see Methods).

Because the V3 domains in Aβ, Prp and HIV-gp120 were reported to have a specific affinity for sphingolipids [22], in particular glycosphingolipids containing a terminal galactose [42], we asked whether SBD would occupy the same membrane domains and follow the same endocytic pathways as previously characterized markers that may also associate with glycosphingolipids. Therefore, we first tested SBD colocalization in human neuroblastomas over time with CtxB, a reagent commonly used as an exogenous marker of glycolipid-containing domains [35].

**Figure 5:**
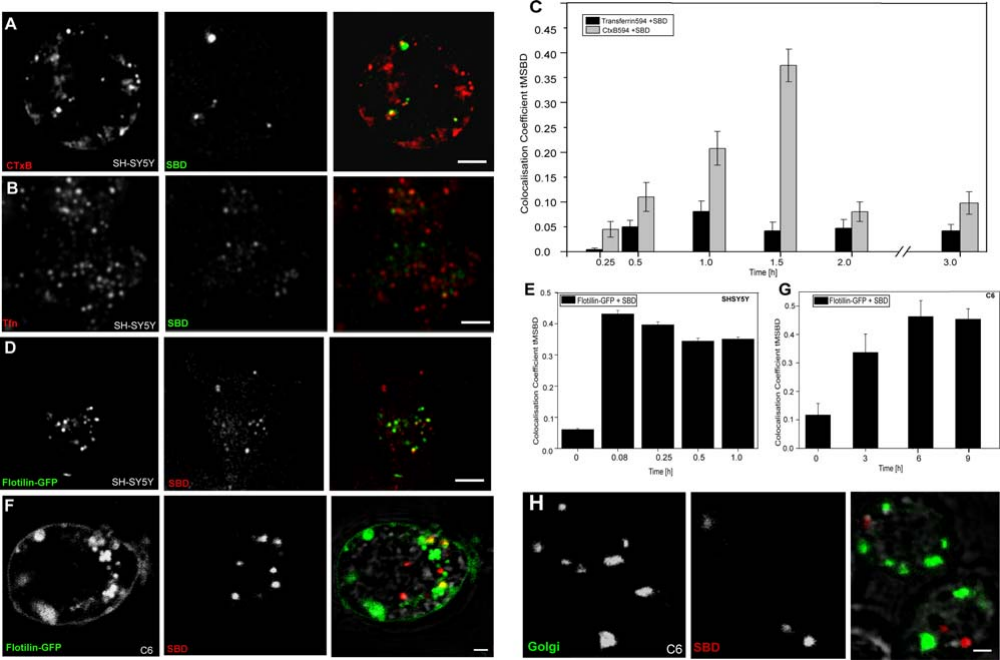
SBD trafficking converges to differing extents over time with lipid raft markers CtxB and Flotillin-GFP, but not with clathrin-uptake marker Transferrin or Golgi. A. Colocalization of CtxB and SBD in SH-SY5Y neuroblastomas. CtxB-Alexa546 (red) was incubated for 30 min in growth medium at 37°C, washed, then cross-linked with anti-CtxB (Vybrant, Invitrogen) for 15 min in growth medium at 37°C, and washed. SBD-OG (green) was incubated immediately thereafter at 5 µM in growth medium for 10 min at 37°C. Both labels were chased in phenol-red-free growth medium and imaged at the time-points given in graph (C). B. SBD does not colocalize with Transferrin (Tfn)-Alexa594, a marker of clathrin-mediated uptake, in SH-SY5Y neuroblastomas. Cells were labelled with Tfn-Alexa594 (10 µg/ml) for 30 min at 37°C, washed, and incubated with SBD-OG (5 µM) for 10 min at 37°C, both in growth medium. Scale bar = 5 µm for A, B, D. C. Colocalization time-course of SBD-OG with CtxB (gray bars) and Tfn-Alexa594 (black bars) in SH-SY5Y neuroblastomas. D. Flotillin2-GFP expressing SH-SY5Y neuroblastomas (green) were labeled 24h after transfection with SBD-TMR as in (A). E. Quantification of Flotillin2-GFP vs. SBD-TMR in SH-SY5Y. F. Flotillin2-GFP expressing c6 cells (green) were labeled 24h after transfection with SBD-TMR (10 µM) in growth medium for 15 min at 25°C. SBD-TMR (red) uptake vesicles are distinct from Flotillin2-GFP vesicles in c6 cells, but increase in colocalization over time (graph in G). Cells were imaged between 60–90 min after application of labels in A–F. Scale bar = 2 µm. G. Quantification of Flotillin2-GFP vs. SBD-TMR in c6 cells. H. SBD shows virtually no accumulation in the Golgi body in c6 cells. C6 cells were labelled as above with SBD-TMR, chased, fixed, and stained with an anti-Drosophila Golgi antibody (Merck). Scale bar = 2 µm.

In order to compare their trafficking routes, fluorescently coupled CtxB-Alexa594 (Vybrant; Invitrogen) was applied after initial incubation with SBD, and chased with fresh medium. Colocalization of CtxB with SBD was minimal at early time points in mammalian neuroblastomas (∼5–10%; fig. 5A, C). After uptake, the SBD-positive vesicles overlapped to a moderate degree with CtxB (35–40% maximum; fig. 5C). Colocalization dropped again precipitously after 1.5h, presumably due to targeting of CtxB to the SBD-negative Golgi body. SBD showed no significant colocalization with a marker of clathrin-mediated uptake, Transferrin-Alexa594 [64], [65] (fig. 5B, C).

The Flotillins are transmembrane proteins that define a subtype of non-caveolar cholesterol-dependent domain [66], [67]. Flotillin2-GFP [39] and SBD in neuroblastoma also colocalized minimally at uptake, and converged at later time points (fig. 5D, E). Similarly, Flotillin2-GFP colocalized initially very little with SBD in c6 cells, but later colocalized substantially (45%; fig 5F, G). Flotillins are taken up and trafficked independently from CtxB and GPI-GFP [40], [68], suggesting that the Flotillins, CtxB, and SBD each occupy different plasma membrane domains. These markers also use distinct classes of GTPases and/or combinations of accessory proteins for their uptake (reviewed in [11]; D. Zhang & R. Kraut, in prep).

CtxB traffics to the Golgi body [35], [69], whereas a substantial portion of both SBD and Flotillin are targeted to late endosomes and lysosomes (see section IV below; [40], [68]). Consistent with this, colocalization between SBD and Flotillin2-GFP is higher than that between SBD and CtxB (fig. 5C, E). In summary, the colocalization data with CtxB and Flotillin-2 indicate that these two labels initially bind to different plasma membrane domains from SBD, but that they subsequently converge to differing extents during sorting.

Lipid microdomains are formed in the Golgi and some proteins that associate with membranes via a GPI-linkage are found in the Golgi [35], [70], [71] (but see [36]). For this reason, we wondered whether the SBD might also associate with the Golgi. SBD showed little or no colocalization with a Golgi-specific antibody in c6 cells (fig 5H).

### IV. SBD traffics via sorting and recycling compartments to late endolysosomes in neurons

**Figure 6:**
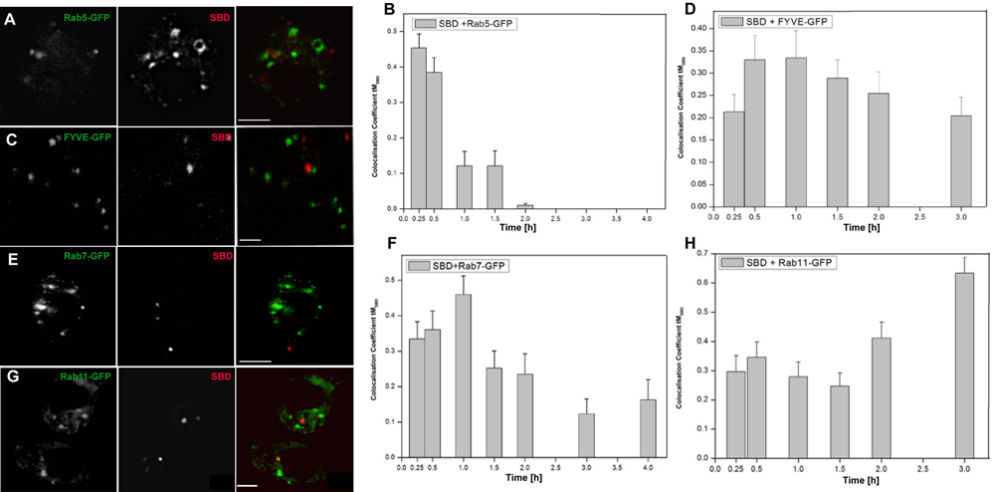
Pulsed SBD-TMR traffics sequentially via rab5-, rab7-, and FYVE-positive vesicles to rab11 recycling endosomes in c6 cells. A, B. SBD colocalizes maximally with rab5-GFP positive early endosomes between 15 and 30 min. Cells were imaged 30 min after application of SBD. C, D. SBD colocalizes maximally with FYVE-GFP positive sorting and multivesicular endosomes between 30 min and 2 h. E, F. SBD colocalization with rab7-GFP positive late endosomes peaks at 1 h. G, H. SBD colocalizes strongly with rab11-GFP positive recycling endosomes only later, at 3 h. For all labellings, SBD-TMR (10 µM in growth medium) was incubated at 25°C for 10 min on cells that had been transfected with the indicated constructs 24–40 h earlier. Cells were imaged at 60 min after application of SBD-TMR in C, E, and G.

After uptake in membrane domains, disparate endocytic cargoes merge in an early sorting endosomal compartment [72]–[75]. As a marker for this sorting domain, we used rab5-GFP expressed in c6 neurons [76]. Shortly after application, SBD-TMR often appeared surrounded by rings of rab5-GFP, indicating uptake into a vesicular sorting compartment (Fig. 6A), consistent with early colocalization values of ∼45% (fig. 6B). SBD colocalized maximally with the later endosomal-to-lysosomal transport marker rab7-GFP [72], [76] at a slightly later time point than rab5-GFP (∼45% at 1h; fig. 6E, F). FYVE-GFP, a marker of sorting endosomes which also localizes to multivesicular endosomes [72], [76], surrounded SBD-TMR in some cases, and colocalization at moderate levels encompassed a longer time frame from a presumptive sorting compartment (30 min; 30–35%) to late endosomal compartments (1.5–2 h; ∼25–30%)(fig. 6C, D). After 3 h, SBD reached very high levels (65%) in a rab11-GFP recycling compartment (fig. 6G, H).

**Figure 7:**
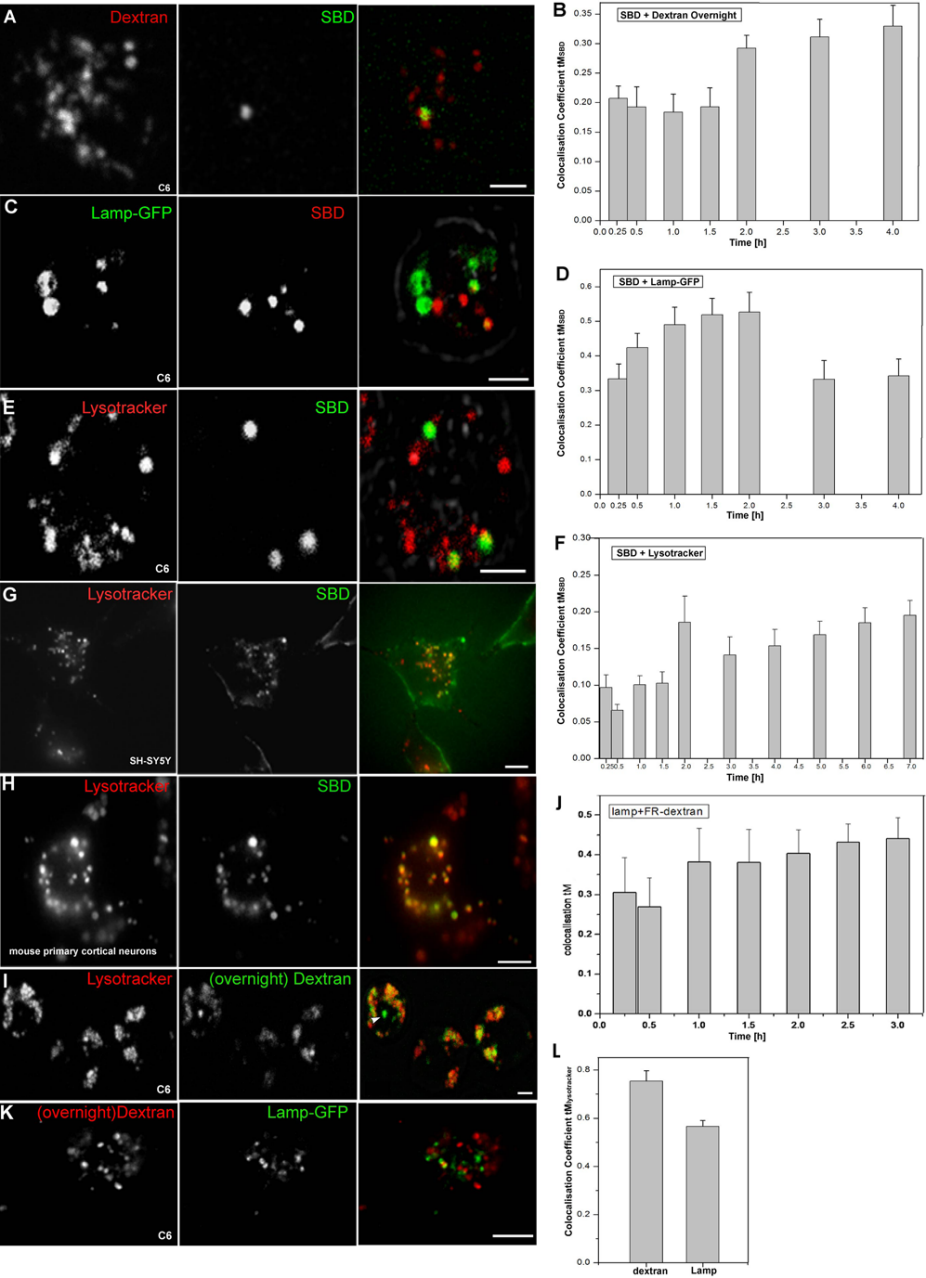
SBD traffics slowly to endolysosomes in c6 cells (A–E) and is found in lysotracker-positive compartments of mammalian neurons (G, H). A, B. Pulsed SBD-OG (green) (incubated at 5 µM in growth medium at 25°C for 10 min) reaches Dextran-Alexa670 (0.5 mg/ml; red) labelled late endolysosomal compartments maximally after 2 h of chase in growth medium. C, D. SBD-TMR (red), incubated at 10 µM in growth medium 24 h after transfection with LAMP-GFP (green) colocalizes maximally in presumptive sorting and late endosomal compartments, up to 2 h of chase in growth medium. E, F. SBD-OG (green) (incubated as in A, B) colocalization with lysotracker (red) (incubated at 75 nM 2 h at 25°C) peaks transiently in a late endosomal compartment (2 h) before reaching a moderate maximum (∼20%) only after 5–7 h (see control graph in fig. 7A). Cells were imaged between 60–90 min after application of SBD in A–E. G, H. SBD-OG (green; incubated at 2 µM for 15 min at 37°C) also colocalizes extensively with lysotracker in SH-SY5Y neuroblastomas, and in mouse primary cortical neurons, shown at 60 min after application of SBD (incubated at 75 nM 2 h at 37°C for both; movie of neuroblastomas shown in fig. S3). I–L. Relative distributions of different endolysosomal markers in c6 cells. (I) Lysotracker (red; labelled as in E) and Dextran-Alexa488 (incubated at 0.5 mg/ml for 5 min at 25°C) chased overnight (green) colocalize ∼75% (L), but some Dextran vesicles are not acidic, indicated by the absence of lysotracker (arrowhead). J. Pulsed Dextran-Alexa670 (incubated as in I, 24 h after transfection) colocalizes with LAMP-GFP throughout its trajectory, reaching maximum tM ∼45% (also see Sriram et al, 2003). K, L. Transfected LAMP-GFP labels both acidic and non-acidic vesicles, as judged by substantial non-colocalization with lysotracker-red (incubated as in E). Scalebar in all images = 5 µm.

Raft-borne sphingolipids such as sphingomyelin and glycosphingolipids can be trafficked to the late endosome/lysosome, where they are broken down [77]–[80]. For this reason, SBD-TMR colocalization with markers of the late endolysosomal pathway was tested. Since TMR is non-pH-sensitive, trafficking to acidic compartments would be detected. As a marker for lysosomal localization of SBD, we used Dextran10kDa-Alexa670 (Invitrogen) incubated overnight. Consistent with Dextran being localized exclusively in lysosomes or late endosomes, colocalization reached its highest level from 2–4 h (30–35%; fig. 7A, B). We also examined colocalization of SBD-TMR with the more broadly distributed endolysosomal marker LAMP-GFP [81], [82]. SBD overlapped extensively with LAMP-GFP throughout the endolysosomal trajectory, beginning in presumptive sorting endosomes and peaking in late endosomes (15min–2h; fig. 7C, D). Notably, the colocalization time-course of SBD with the acidic compartment marker Lysotracker Red was low and slow compared to the other endolysosomal markers, peaking after only 6–7 h at ∼20% (fig. 7E, F).

In order to confirm SBD's localization to endolysosomes in different neuronal cell types, human neuroblastoma SH-SY5Y and mouse cortical neurons were labeled with Lysotracker and pulsed with SBD-OG, showing extensive overlap (fig. 7G, H [not quantified]; movie shown in fig. S4).

The relative localizations of the different available markers of late endolysosomal/acidic compartments were compared by incubating Lysotracker on cells whose lysosomes had been overnight labelled with Dextran-Alexa488 (Dextran o/n), or transfected with LAMP-GFP and pulsed with Dextran. As expected, lysotracker showed a high tM with Dextran o/n (75.3% ±4.3%), whereas the tM of lysotracker with LAMP-GFP was lower (56.5% ±2.5%) (Fig. 7I–L). LAMP-GFP colocalized broadly with pulsed Dextran throughout its trajectory (fig. 7J). This suggests that of the three lysosomal labels, LAMP-GFP has the widest distribution spanning both late endolysosomal and sorting compartments.

In summary, the SBD colocalization time-courses indicate that like caveolin, but unlike the GEEC pathway (GPI-anchored-protein-enriched Early Endosomal Compartment) [6] SBD is sorted through a rab5-GFP-positive early endosomal compartment. Thereafter, a portion of SBD traverses rab7-GFP-positive late endosomes and may travel through FYVE-associated multivesicular bodies, as has been reported for Aβ [83]. Finally, a large part of the SBD pool (∼60%) reaches a recycling compartment at a late timepoint (between 2–3 h), and the remainder localizes to lysosomes labeled by Dextran.

### V. SBD traffics similarly to Dextran and lactosyl ceramide

**Figure 8:**
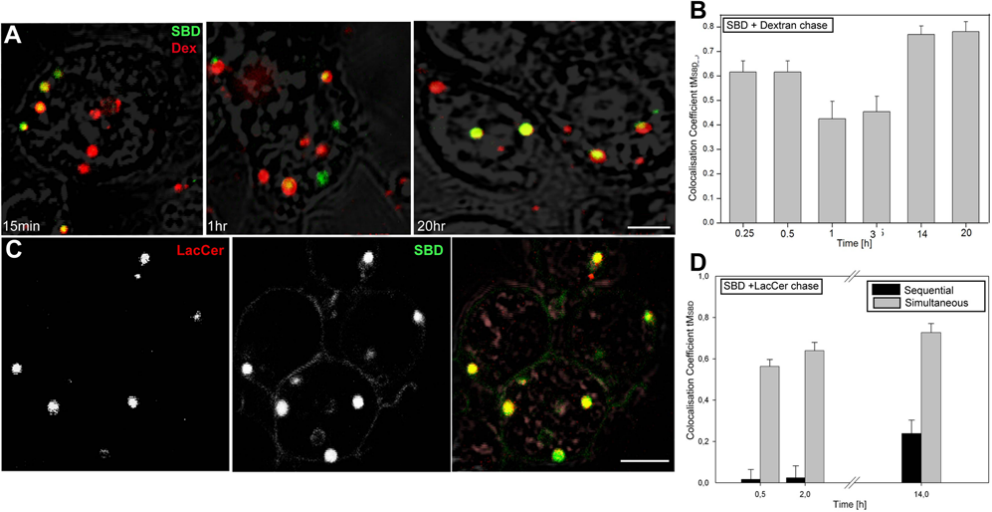
SBD-OG traffics similarly to pulsed Dextran-Alexa670 and lactosyl-ceramide when both labels are present simultaneously at the plasma membrane. A, B. SBD-OG follows a similar pathway to Dextran-Alexa670 (shown after 15′, 1 h, and 20 h), but appears to diverge transiently in post-sorting late endosomes between ∼60 min and 3 h (see movie S3). Cells were incubated first with SBD-OG at 5 uM in HBSS for 15 min at 25°C, washed once, and incubated in Dextran-Alexa670 at 0.5 mg/ml in HBSS for 5 min, washed, chased in HBSS, then imaged at the time-points given in the graph (B). C, D. BODIPY-lac-cer (red) shows nearly complete colocalization with SBD-OG (green) when incubated simultaneously on c6 neurons (gray bars; see Methods). Sequential incubation (black bars) leads to much lower colocalization scores. Even after 14 h, colocalization levels never approach those attained with simultaneous incubation, indicating an altered trafficking route taken by at least one of the two labels. For simultaneous SBD/lactosyl-Ceramide labellings, cells were incubated with lactosyl-Ceramide (5 mM at 4°C for 30 min, washed once, incubated with SBD-OG (25°C as in A), chased in HBSS, and imaged at the time-points given in graph (D). For sequential labellings, cells were incubated with BODIPY-lac-cer at 25°C, washed several times in warmed HBSS to allow uptake, then incubated with the second label (SBD-OG) at physiological temperature (concentrations as above), washed in HBSS, and chased in growth medium. Scalebar = 5 µm.

We tested SBD colocalization over time with pulse-chased Dextran10kDa-Alexa670 (Dextran uptake in Drosophila haemocytes has been described [84]), which at low concentrations is incorporated into cdc42-dependent, non-clathrin associated endocytic vesicles en route to lysosomes [36], [84], [85]. SBD colocalization with Dextran is high (∼60%) in presumptive early endosomes at 15′, drops in late endosomes (∼40%) at 1–3 h, and rejoins Dextran in a presumptive lysosomal compartment thereafter (∼80%) (fig. 8A, B). Corresponding to a tM value of 0.8, this is close to the maximum seen in completely colocalizing controls (e.g. SBD-TMR+SBD-OG; not shown). The dip in colocalization with Dextran at 1–3 h corresponds to a sequential rise in colocalization with rab7 and rab11 (fig. 6F, H). After diverging from the Dextran pathway at 1–3 h, SBD then converges with Dextran in presumptive lysosomes (see fig. 8B; see model, Fig. 7). A time-lapse movie taken 90 min after c6 neurons were labeled with both Dextran and SBD demonstrates this phenomenon as SBD-carrying vesicles can be seen fusing with Dextran-positive vesicles (fig. S5).

Several fluorescent sphingolipid analogs have been used as tracers of endocytic trafficking pathways in cellular models of lipid storage diseases [12]. Although N-acyl substituted analogs do not behave in a manner completely analogous to endogenous sphingolipids [86], [87], certain species can be used as diagnostic markers for trafficking defects [64], [73], [78], [88], [89]. Fantini and colleagues have postulated that the V3 loop could interact with the terminal galactose of glycosphingolipids and with sphingomyelin in raft domains [1], giving us reason to think that SBD might interact more strongly with lactosyl-ceramide (gal-glc-Cer; lac-Cer) or sphingomyelin than with other sphingolipid analogs. Therefore, we were interested in testing the behavior of SBD with respect to these markers.

In our hands, only fluorescent ceramide and lac-Cer analogs were endocytosed by c6 cells. (A characterization of fluorescent lac-Cer and ceramide trafficking in Drosophila cells will be reported elsewhere (RH, EL, SH, SS and RK, submitted). SBD showed the strongest colocalization of any marker throughout its endocytic trajectory with BODIPY-lac-Cer (tM_SBD_ 70%_;_ fig. 8C, D), which remains intact in c6 cells for up to ∼14 h (ibid). Strikingly, this co-trafficking of SBD and lac-Cer was only observed when the two labels were present at the membrane simultaneously, but not when they were added sequentially (fig. 8B, “simultaneous” vs. “sequential”; see Methods). The effect was not observed with Dextran, where SBD colocalization was similar after simultaneous or sequential addition (not shown). Lac-Cer has been reported to stimulate caveolar uptake [90], so the observed strong colocalization of lac-Cer with SBD could reflect a stimulation of endocytic uptake that carries both labels. However, the fact that SBD never catches up with lac-Cer even after 14 h suggests that a different mechanism is involved, perhaps a specific interaction between SBD and the glycosphingolipid that influences the trafficking pathway of one or both labels.

### VI. SBD trafficking to lysosomes depends on cellular cholesterol content

The formation of lipid microdomains, defined as more or less transient regions of liquid ordered, saturated lipids in the membrane, are thought to involve specific associations between sphingolipids and cholesterol [4], [88], [91]. In keeping with SBD's interaction with membrane domains that depend on the presence of cholesterol, we showed previously that its uptake is less efficient and its diffusion at the membrane is faster after cholesterol depletion with methyl-β-cyclo-dextrin (MβCD) [51]. The intracellular trafficking of sphingolipid analogs is also known to be affected by cholesterol overloading of mammalian fibroblasts and neurons [12]. We therefore reasoned that SBD trafficking might be altered by cholesterol depletion or excess if it is indeed acting as a sphingolipid tracer. However, it was not clear that this would be the case in Drosophila, since flies are cholesterol auxotrophs. In order to test this, we depleted Drosophila c6 cells of cholesterol and related sterols with MβCD treatment (see Methods), and looked at the effect on SBD trafficking with respect to lysosomal and endosomal markers. The effectiveness of the MβCD depletion and cholesterol overload was confirmed (40% reduction in total cholesterol after MβCD depletion; 250%, ±13% increase after overload; see [51]). SBD trafficking in mammalian neurons is also affected by cholesterol manipulations, and will be presented elsewhere (DZ, GT and RK, in prep).

**Figure 9:**
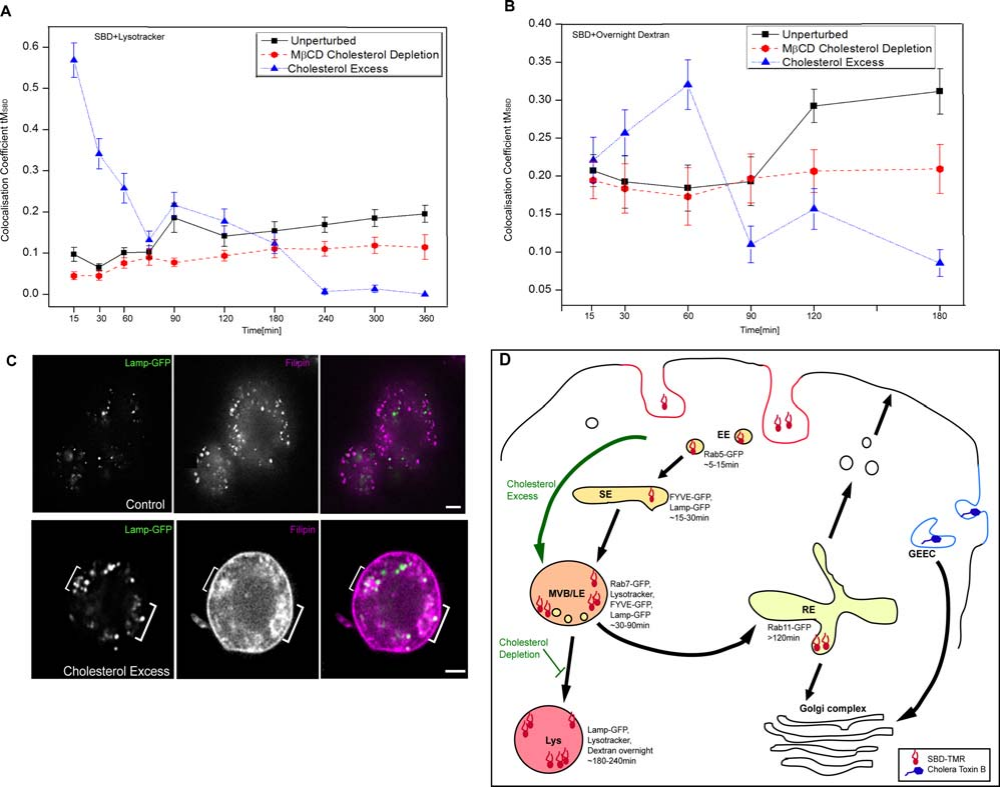
SBD trafficking to acidic organelles is sensitive to cholesterol levels. A. C6 cells depleted of cholesterol with MβCD (10 mM 30 min 25°C; red circles) or treated with excess cholesterol (10 mM MβCD-10mM cholesterol complexes for 30 min 25°C; blue triangles), or untreated (black squares), then labeled with lysotracker and pulsed with SBD-OG. Colocalization (tM_SBD_) was calculated at the given time points of chase. Data for control untreated cells were compiled from two experiments. B. C6 cells were labeled with Dextran-Alexa488 overnight, and then depleted of cholesterol (red circles), or treated with excess cholesterol as in (A)(blue triangles), or untreated (black squares) and labeled with SBD-TMR, as in fig. 5F. Colocalization was calculated at the given time points of chase. C. Control c6 cells (top panels), or c6 treated with excess cholesterol (bottom panels) after transfection with LAMP-GFP (green) and subsequent fixation show heavy accumulations of filipin (pink) at the plasma membrane and near, but not exclusively in, endolysosomal compartments (brackets). Total cholesterol content after cholesterol overloading was increased by 250%, ±13% (n = 3; quantification by Amplex Red enzymatic assay not shown). Scale bars for all images = 5 µm. D. Schematic representation of the internalization pathway of fluorescent SBD (red shapes) vs. another lipid-raft associated cargo, CtxB (blue hexagons), which is taken up by the GEEC pathway [11]. Cholesterol depletion moderately inhibited transport of SBD through a Dextran-positive lysosomal compartment (>90 min in graph B), whereas cholesterol excess led to premature accumulation and then clearing of SBD from lysotracker- and Dextran-positive acidic compartments. Detection of myc immunoreactivity indicates that internalized SBD-myc has not been degraded. Dot blots were also exposed to anti-β-tubulin as a loading control to ensure that protein amounts were comparable in the various sets. Intensity of dots was quantified using Quantity One software and represented in bar graphs.

SBD that was taken up in the cholesterol depleted c6 cells was found to be trafficked to lysosomes somewhat less efficiently, based on a lower than normal colocalization profile with Dextran-labelled lysosomes, particularly at later time points (fig. 9A, B). Under cholesterol overload (shown by filipin staining in and around endolysosomal compartments; fig. 9C) SBD was actually endocytosed more readily (quantification in fig. S6), but trafficking was strongly perturbed. After uptake by cholesterol-loaded cells, SBD was prematurely and abnormally trafficked to an acidic lysotracker-positive compartment, and to Dextran-positive lysosomes, after only 15 min and 1 h, respectively. Colocalization with both of these lysosomal markers dropped precipitously thereafter to levels well below normal. This suggests that cholesterol excess at the membrane and in endolysosomal compartments labelled by LAMP-GFP (fig. 9C) may shunt SBD away from a sorting compartment prematurely into acidic vesicles, and thereafter divert it away from the lysosomal pathway.

## Discussion

This report presents the characterization of a fluorescently tagged peptide probe, the SBD, for tracing the trafficking pathways of sphingolipid-containing membrane microdomains in living cells. We present evidence that SBD binds to artificial membranes with raft-like composition, with improved binding efficiency at low pH and in the presence of gangliosides. Quantitative time-course colocalizations based on the method of Costes [62] are used to describe the endocytic trafficking pathway of fluorescently tagged SBD, and compare its intracellular trajectory with those of other presumptive microdomain-specific and endolysosomal markers.

Consistent with the model of Fantini et al, wherein aromatic and basic residue(s) in the SBD were proposed to interact with galactose-terminal glycolipids and sphingomyelin, we found that SBD-TMR interacted optimally with the combination of POPC/SM/Chol, and the strongest enhancement of binding was seen when ganglioside was added to this mixture. The ability to bind POPC/SM/Chol may be partially attributable to the tendency of this mixture to phase separate into liquid ordered domains (confirmed by atomic force microscopy [GT & RK, unpublished results]; [58]), since a saturated glycerophospholipid with the same choline headgroup (SPPC), which would also be expected to phase separate, was able to substitute for sphingomyelin to some extent. However, SBD binding to the SPPC mixture was not as high as that which was seen with sphingomyelin. Moreover, addition of glycosphingolipid to the SPPC mixture abolished binding, whereas it strengthened the binding of the sphingomyelin mixture. Therefore, although an interaction with other PC-containing liquid ordered domains is possible, it appears likely that under conditions at the plasma membrane of a cell, where glycolipids are present, a PC-headgroup sphingolipid (i.e. sphingomyelin) is strongly preferred.

SBD showed a preference for multiply sialylated gangliosides at neutral pH, similarly to amyloid peptide Aβ1−40 [47], [49], but the specificity for a particular ganglioside is less strict than is the case with CtxB. Interestingly, the strength of SBD's interaction with ganglioside-containing membranes is greatly enhanced at low pH. Because of this, one can speculate that as SBD is endocytosed and the pH drops, its interaction with the membrane may be strengthened by masking of electrostatic repulsion between SBD (which has a pKa of ∼4.25) and sialic acid. Given the overall negative charge of SBD, however, its predilection for GT1b, GQ1b, and GD1a (which have multiple sialic acids) over GM1 (which has one) at pH 7 is difficult to explain. Possibly, the positively charged Arg and His residues near the aromatic Tyr residue proposed by Fantini to be important in binding via π-bonding interactions [1], actually attract SBD to more heavily sialylated gangliosides. This would be supported by the work of Ariga et al [47], and by our experiments at neutral pH. On the other hand, SBD's preference for less sialylated glycolipids (e.g. GM1) in acidic endosomes suggests the possibility that Fantini's proposed π-bonding with the exposed galactose, or a specific structural interaction with terminal galNAC-gal-sialic acid, may begin to dominate when the charges are neutralized.

Confirming the spectrofluorimetric liposome binding assays above, SPR analysis showed improved binding of SBD-TMR to the same liposomes immobilized on a Dextran-coated gold chip, as the concentration of GD1a was increased. However, we note that binding of SBD reacted relatively weakly and gradually to low concentrations of ganglioside, and required up to 20% to give the maximal response, as compared to CtxB, which bound very strongly already at just 5% GM1. These results together with the lack of intracellular colocalization with CtxB (below) suggest that recognition of different glycolipids (in this case GD1a vs. GM1), perhaps in combination with membrane proteins, may mediate uptake into distinct intracellular trafficking routes, as suggested by Lencer and colleagues [92].

As would be expected for a raft-binding probe [1], SBD showed no detectable colocalization with Transferrin, which is internalized by clathrin-mediated endocytosis [74]. Unexpectedly, however, SBD showed only low to moderate overlap upon uptake and trafficking in neuroblastomas with the sphingolipid-interacting marker CtxB. In some mammalian cell types, CtxB is thought to follow a cdc42-dependent uptake pathway [36], [40], [72], [93] but it can also be endocytosed by a mixture of non-clathrin and clathrin-mediated mechanisms in neurons [28]–[30], [93], [94]. SBD showed higher colocalization with another raft-localized marker, Flotillin2-GFP which, like SBD, traffics to a large extent through the endolysosomal pathway [40], [68].

The non-colocalization with CtxB was all the more surprising, because of reports that Aβ (from which SBD is derived) binds to the same glycosphingolipid as CtxB, namely GM1 [21], [95], [96]. Aβ, however, can also bind to other glycosphingolipids, dependent on conformation [47], [49]. From these results we conclude that SBD is internalized by a non-clathrin pathway that is distinct from other cholesterol-dependent pathways, reflecting the probable heterogeneity of lipid microdomains as has been noted in many other studies [10].

After uptake, SBD overlapped extensively with the endolysosomal tracer Dextran, which at low concentrations is endocytosed via a cdc42-dependent uptake pathway [40], [85], [93]. Early, SBD trafficked through a rab5→FYVE→rab7 trajectory, finally reaching lysosomes after 3–4h (summarized in fig. 9D). A significant amount (tM ∼60%) of SBD also trafficked from late endosomes (1.5–2 h) to a rab11-GFP-positive recycling compartment, which has been reported to be particularly rich in sphingomyelin and cholesterol in mammalian cells [26], [77], [97], [98]. These findings are interesting with regard to the discovery that mutations in Alzheimer's disease-linked SORL1 result in shunting of App away from recycling endosomes toward the endolysosomal pathway [99], [100]. More experiments will be required to determine whether and how App trafficking and SBD trafficking are related.

Of all the markers tested, the highest degree of colocalization was found between SBD and the glycosphingolipid analog BODIPY-lac-Cer. Interestingly, this was only true when the two labels were present at the membrane simultaneously; sequential addition resulted in nearly no colocalization. The finding that SBD and lac-Cer trafficked so closely together was surprising, since lac-Cer's trafficking itinerary is different from that of SBD in both mammalian [78], [101] and Drosophila cells (RH, EL, SH, SS & RK, submitted). Their co-trafficking may result from SBD's affinity for glycosphingolipids, or may reflect a phenomenon we have documented in a separate study, termed “hijacking”, whereby one label influences the trafficking behavior of the other. The hijacking mechanism is apparently different from the simple stimulation of uptake reported by Pagano and colleagues [90], since we find that even over extended chasing times, the second label added sequentially never reaches the same compartment as the first, which would be expected if only endocytic stimulation were responsible (RH, EL, SH, SS & RK, submitted).

The interaction of SBD with glycosphingolipids is consistent with our finding that its trafficking to lysosomes is strongly affected by cholesterol levels, since glycolipids are also known to traffic aberrantly in cholesterol storage disease fibroblasts [12]. Paradoxically, however, we find that SBD trafficking to the lysosome is ultimately diminished by excess cholesterol, in contrast to glycosphingolipids, which accumulate to excess in endolysosomes under similar conditions. In summary, it appears that SBD trafficking is strongly influenced by cellular cholesterol levels, especially cholesterol overload, and this may reflect an underlying event involving the trafficking of its glycosphingolipid partner(s). Moreover, changes in SBD targeting may provide insights into the effects of cholesterol perturbation on the trafficking behavior of Aβ, since their binding characteristics appear to be similar.

### Conclusion

In this study, we describe the intracellular trafficking of a novel fluorescent sphingolipid-interacting marker, the SBD, and present evidence that it can interact with raft lipids sphingomyelin, cholesterol, and glycosphingolipids, but without a highly specific interaction to a particular ganglioside, like CtxB. Thus, SBD can potentially be used to trace the intracellular pathways of sphingolipid-containing domains, including at least some gangliosides. There is little doubt about the utility of being able to follow the trafficking routes of sphingolipids and microdomains in living cells, yet very few non-transgenic probes are currently available that track these lipids.

The SBD probe is a short, non-toxic peptide, and can therefore be produced easily as a fluorophore-linked conjugate and applied exogenously to cells. Together with the observation that SBD's trafficking route reacts strongly to cholesterol perturbation, these properties make it well suited for applications in diagnostic and drug screening assays. Additionally, the fact that SBD behaves similarly in Drosophila and mammalian neurons validates its use as a tool for studying lipid trafficking disease models in the fly. Further characterization of the behavior of the SBD in the context of genetic and lipid perturbations will allow the use of this probe in models of neurodegenerative and other diseases that are linked with the metabolism and trafficking of sphingolipids. Information gained from such studies would contribute to our understanding of the involvement of sphingolipid trafficking in neurodegenerative disease pathologies.

## Materials and Methods

### Cell culture

Growth media: Drosophila neuronal cell lines DL-DMBG2-c6 (Drosophila Genome Resource Center; Ui et al, 1997) were grown at 25°C in Schneider's medium (Gibco, USA) with 10% fetal bovine serum (FBS; Gibco, USA), 0.125IU/ml bovine insulin (Biological Industries, Israel), and 1% antibiotic/antimycotic solution (Gibco, USA). NIH3T3 mouse fibroblasts and SH-SY5Y neuroblastoma (ATCC, USA) were grown at 37°C in Dulbecco's Modified Eagle's Medium (DMEM; Gibco, USA) and DMEM/F12 (Gibco,USA)respectively, both with 10% FBS and 1% antibiotic.

Rat and mouse embryonic cortical neurons were prepared using the papain disassociation technique described previously [55] and cultured on 8-well, coverglass-bottom dishes for 3–7 days prior to analyses.

### Production of fluorescently tagged SBD peptide, cell labelling, and degradation assays

SBD peptide preceded at the N-terminus by two copies of an inert spacer ([AEEAc]2) was conjugated to Oregon Green (OG) via a thiol linkage to an N-terminal Cysteine, or Tetramethylrhodamine (TMR) via an amide linkage directly to the spacer, was synthesized by Bachem, Switzerland, such that the final sequences were OG-Cys-[AEEAc]2-DAEFRHDSGYEVHHQELVFFAEDVG, and TMR-[AEEAc]2- DAEFRHDSGYEVHHQELVFFAEDVG, respectively. A mutated sequence (DAEF**A**HDSG**A**EVHHQELVFFAEDVG) and a scrambled sequence (FYHDESEFGHAVEQFGRDVEAVHDL) were also coupled to the fluorophores and to myc as controls. To avoid aggregate formation of the peptide, SBD was dissolved in 1,1,1,3,3,3-Hexafluoro-2-propanol (HFIP) (Lancaster, UK), aliquoted and dried. Lyophilized peptide was stored at −20°C, and redissolved immediately before use in DMSO. Peptide was diluted to a final working concentration of 10 µM in Hanks Buffered Salt Solution (HBSS; Gibco) supplemented with 10 mM Hydroxyethylpiperazine-ethanolsulfonic acid (HEPES) or in growth medium, and incubated at 25°C for 15 min at 10 µM or 5 µM (for SBD-TMR and SBD-OG respectively, on c6 cells) or 37°C at 2 µM (for mammalian cells), and then washed three times in HBSS/HEPES (for double labellings with lipid analogs), or growth medium (for double labellings with other markers).

Conjugation of SBD was confirmed by HPLC and mass spectrometry by the manufacturer (Bachem AG, Bubendorf, Switzerland); HPLC and MS peaks showed no change in structure of the peptide, and elution at the expected molecular weight. Additional confirmation of the conjugation efficiency of SBD to fluorescent labels was carried out by fluorescence correlation spectroscopic (FCS) measurement of diffusion times of the uncoupled fluorescent label diluted to ∼1 nM. A confocal Zeiss Axiovert 200 microscope combined with Igor Pro Software (Wavemetrics) was used for FCS measurements, which were run in multiples of 5 sets each lasting for 30 sec. The obtained correlation functions were fitted, corresponding parameters calculated and finally averaged.

To test whether SBD was degraded after uptake in cells, SH-SY5Y neuroblastoma were incubated with 5 µM Myc-tagged SBD (synthesized by GenScript Corp, New Jersey) for 10 min, and then replaced with fresh medium. After 10, 30, and 60 min chase, cells were lysed in Tris-NaCl, EDTA buffer with 0.5% Triton-X100. Lysates with equal amounts of protein (estimated by BCA assay) were blotted onto nitrocellulose membranes for dot blots. The same procedure was carried out for peroxidase-conjugated CtxB (1 µg/ml) as a control. After blocking, membranes were exposed to peroxidase-conjugated anti-myc (Santa Cruz) and blots were developed using chemiluminescence.

### Cell labelling with fluorescent lipid analogs and endolysosomal tracers

Cells were seeded at a density of 10^6^ cells/ml into either 8-well chambers with 0.17 mm coverslip bottoms (Nunc, Denmark) or 25 mm dishes with coverslip bottoms (Fluorodishes; WPI). Experiments were conducted 24–72 h post-seeding. Before addition of fluorescent lipid analogs, cells were washed 3 times with HBSS/HEPES, unless otherwise stated. The following lipid analogs with attached 4,4-difluoro-4-bora-3a,4a-diaza-s-indacene (BODIPY) fluorophore were from Molecular Probes (Invitrogen): BODIPY-C5-ceramide, BODIPY-FL-C12-Sphingomyelin, BODIPY-FL-C5-lactosylceramide. Cells were washed with pre-chilled HBSS/HEPES, incubated with 5 µM lipid for 30 min at 4°C, washed with ice-cold HBSS/HEPES and chased for various times with fresh growth medium supplemented with 1% Oxyrase (Oxyrase, Inc., USA) at 25°C or 37°C.

Lysotracker staining of acidic compartments was done by incubating cells for 2 h with 75 nM Lysotracker Red (Invitrogen) in normal growth medium.

Alexa594- or Alexa546-coupled Cholera Toxin B (CtxB) (Vybrant kit; Invitrogen) was incubated at 5 µg/ml in growth medium for 30 min at 37°C on neuroblastoma cells, washed, and cross linked with anti-CtxB antibody provided in the kit (5 µg/ml in growth medium) for 15 min at 37°C. For double labelling with SBD-OG, at this point 5 µM SBD in growth medium was added for 10 min at 37°C, washed, and imaged at various time-points after chasing with 37°C growth medium.

For double labellings of neuroblastoma cells with transferrin (Tfn), Tfn-Alexa594 (Invitrogen) was incubated in growth medium at 10 µg/ml for 30 min at 37°C, washed, and incubated in SBD-OG at 2 µm/ml for 10 min in growth medium, washed, and chased in growth medium.

For pulse labelling c6 cells with Dextran, cells were incubated for 5 min at 25°C with 0.5 mg/ml Alexa488- or Alexa670-Dextran (10,000MW; Invitrogen) in HBSS/HEPES, washed with HBSS/HEPES, and imaged after various chasing times.

### Simultaneous vs. sequential labelling

For SBD/Dextran double labellings described as “simultaneous”, the cells were incubated with SBD for 15 min, washed quickly once, then incubated with Dextran for 5 min, chased with fresh medium and imaged immediately. For simultaneous SBD/lactosyl-Ceramide labellings, cells were incubated with lactosyl-Ceramide at 4°C for 30 min, washed once, incubated with SBD for 15 min in HBSS at 25°C such that both labels would be present at the plasma membrane simultaneously at the beginning of the time-chase with fresh growth medium. For “sequential” labellings, cells were incubated with the first label at physiological temperature, washed several times in warmed HBSS to allow uptake, then incubated with the second label at physiological temperature, washed in HBSS, and chased in growth medium.

### Production and labeling of liposomes

Liposomes were prepared from a ternary mixture of 45% 1-palmitoyl-2-oleoyl-sn-glycero-3-phosphocholine (Avanti Polar Lipids, Alabaster, AL), 25% sphingomyelin (bovine brain; Sigma) and 30% cholesterol (Sigma). Bovine brain derived monosialoganglioside GM1 (Avanti Polar Lipids) and GD3 (Accurate Chemical & Scientific, Westbury, NY), disialoganglioside GD1a (Sigma), trisialoganglioside GT1b (Sigma), and tetrasialoganglioside GQ1b (Accurate Chemical & Scientific) were incorporated into the initial lipid mixture in the desired ratios. The lipids were dissolved in chloroform: methanol, 2:1 (v/v), dried by vacuum rotary evaporation in a round-bottom flask partially submerged in a 42°C water bath subsequently dried overnight in a vacuum chamber. The dry lipids were hydrated in HBSS/HEPES for 2 h at 60°C to 0.5 mg/ml, reduced to ∼100 nm average diameter by sonication. and filtered through a 0.2 µm polycarbonate. This liposome solution was stable at 4°C for several months.

SBD-TMR or CtxB-Alexa594 was added to 500 µL, 0.1 mg/mL liposome solution and incubated for 30 min in Omega Nanosep 300K MWCO centrifugal devices (Pall Life Sciences, Ann Arbor, MI). The unbound SBD peptides were separated by 1 h centrifugation at 10,000 g. Liposomes with bound peptides were resuspended in 500 µL HBSS/HEPES and fluorescence measured with Fluorolog spectrofluorometer (Horiba Jobin Yvon, Edison, NJ) at 543 nm excitation for TMR and 594 nm for Alexa.

### Surface Plasmon Resonance

All measurements were carried out at 25°C in 10 mM HEPES, pH7.4, 150 mM NaCl buffered saline. L1-sensorchip surface was cleaned by an injection of 40 mM 3-[(3cholamidopropyl) Dimethylammonio]-1-propane sulfonate (CHAPS, Ambresco), followed by 20 mM n-Octyl β-D-glycopyranoside (Sigma) and CHAPS. The cleaned Dextran-surface was coated with 50 µL liposome solution at a flow rate of 2 µL/min. Unbound liposomes were removed with a short pulse of 10 mM NaOH. 5 µL of 1 mg/mL BSA was injected to block unbound Dextran. After rinsing the surface, SBD or CtxB was injected into the flow cells at 10 µL/min at 20 µM and 0.2 µM, respectively. The chip was regenerated with 80 µL 40 mM CHAPS.

### Transfection of neuronal cells and DNA constructs

The following DNA constructs were used: pUAST-GFP-LAMP; Flotillin2-GFP was excised from pAc5.1(A)Flotillin2-EGFP [39] and subcloned into pCDNA3.1 (Invitrogen). Transfection was performed using Lipofectamine 2000 (Invitrogen) as per manufacturer's instructions, and subsequent labelling and imaging was carried out between 24–40 h post-transfection.

### Cholesterol depletion and overload

Cells were incubated in 10 mM Methyl-β-cyclo-dextrin (MβCD) (Sigma, USA) for 30 min in serum-free medium, and washed. Cholesterol concentration in cell extracts was measured using Amplex Red Cholesterol Assay kit (Invitrogen), and normalized to protein concentration. For cholesterol excess, 10 mM MβCD:10 mM cholesterol complexes were prepared as in Klein, et al[102] and incubated with cells at 25°C for 30 min before labelling. Treated cells were further incubated in complete medium with FBS.

### Immunocytochemistry and cell viability assays

The following antibodies were used: anti-Drosophila Golgi (Merck), 1∶250; Alexa488, 568, or 633-coupled secondaries (Invitrogen). Cell viability was confirmed using Sytox Green (Invitrogen) as per manufacturer's instructions.

### Imaging and Image processing

Confocal images were obtained on Zeiss LSM510, Leica TCS SP2, and Olympus FV300 microscopes with a 63x/1.4NA oil objective (LSM510, TCS SP2), 60 X/1.4NA oil objective (FV300), or 63x/0.9NA dipping lens (TCS SP2). For double labelling, each fluorescent dye tested for crosstalk into other channels, and different channels were acquired sequentially, with a pinhole diameter of 1 airy unit. Movie in fig. S3 was acquired with a CoolsnapHQ CCD camera on a Deltavision (Applied Precision) widefield microscope with a 60X/1.42NA oil lens (Olympus) and a standard (green ex490/20, em528/38; red ex555/28, em617/73) filter set (Chroma).

SigmaPlot (Systat) and Origin were used to create charts and graphs and figures were assembled in Adobe Photoshop 7.0.1 (Adobe Systems, Inc). Image analysis was performed using ImageJ (rsb.info.nih.gov/ij) with the plugins: “Colocalization Test” and “Colocalization Threshold” by T. Collins and W. Rasband, “BG Subtraction from ROI” by M. Cammer and T. Collins (www.uhnresearch.ca/facilities/wcif/imagej). For live/dead assays cells were counted manually using the ImageJ “Cell count” plugin.

Colocalization was quantified using the thresholding algorithm of Costes et al [62] on background corrected images. Randomizations were done with 25 iterations (Colocalization Test plugin). If colocalization test resulted in no significant difference between randomized and original images (P<0.95), no colocalization was assumed. If colocalization was significant (P>0.95) the second plugin “Colocalization threshold” was applied to determine colocalization parameters. Colocalization was expressed as tM_SBD_ (a fraction between 0 and 1), the Manders coefficient for the SBD channel calculated with the thresholding algorithm of Costes et al. Each data point consisted of two separate experiments, taking into account at least 10 cells each.

## Supporting Information

Figure S1 SBD treatment of cells does not affect cell viability at working concentrations (2–10 µM). Percentages of Drosophila c6 neurons negative for Sytox Green (Invitrogen) are shown for cells labeled with SBD (black bars) or SBD* (gray bars) at the indicated concentrations.
(0.21 MB DOC)

Figure S2 SBD is detectable by dot blots after 60 minutes of chase after uptake. SBD-myc levels in cell lysates of SH-SY5Y neuroblastoma after 10, 30, and 60 minutes of post-incubation chase are comparable on dot blots to levels of CtxB-peroxidase after similar labelling and chase in the same cell line. Quantification of myc intensity levels (see graph) indicates that 85% of SBD-myc is detectable after 60 minutes chase. Intensity levels are normalized to tht after 10 minutes chase. 86% of CtxB is detectable 60 minutes post-incubation. Detection of myc immunoreactivity indicates that internalized SBD-myc has not been degraded. Dot blots were also exposed to anti-β-tubulin as a loading control to ensure that protein amounts were comparable in the various sets. Intensity of dots was quantified using Quantity One software and represented in bar graphs.
(3.21 MB TIF)

Figure S3 Titration curves used to calculate the binding affinity (K_D_) of SBD to raft-like membranes. Response signals of SBD peptide injected at various concentrations between 0.5 µM and 50 µM over a surface homogeneously covered with POPC/SM/Chol+10% GD1a liposomes. Best fit was obtained with a heterogeneous ligand model (black curves). The bulk refractive index (RI) was calculated separately contributing different bulk shift effects at the beginning and end of the injection.
(6.03 MB TIF)

Figure S4 Movie showing SBD (green) incorporation into motile endolysosomal compartments of SH-SY5Y neuroblastomas labeled with Lysotracker (red). Movie was taken by widefield fluorescence microscopy, and spans 5.7 min of real time, 30 min after SBD labelling (2 µM, 15 min, 37°C), at 1.2 sec/ frame, and assembled at 30 fps.
(4.41 MB MOV)

Figure S5 Movie showing SBD-OG (green; incubated for 15 min at 5 µM, 25°C)) and Dextran-Alexa670, 10kDa (blue) in Drosophila c6 neurons after 90 min chase, when colocalization drops to a moderate level (∼40%). SBD-carrying vesicles can be seen fusing with Dextran-carrying vesicles in three of the cells shown, which may represent fusion in late endolysosomal compartments. Individual confocal fluorescence images were collected every 1 sec over 1 min 15 sec of real time, and assembled at 30fps.
(0.07 MB MOV)

Figure S6 Uptake of SBD-TMR (incubated at 10 µM, 15 min, 25°C) after cholesterol overloading of c6 cells with MβCD-cholesterol complexes, assessed by measurement of total intracellular fluorescence intensity in 25 cells per time point up to 2 h after labeling, and expressed as average fluorescence intensity per pixel (quantitation was done as in Cheng et al (Cheng et al., 2006). Uptake is slightly increased in cholesterol overloaded cells (gray bars) over control cells (black bars).
(0.60 MB DOC)
