# Supplementary figures and images for: A Fluorescent Glycolipid-Binding Peptide Probe Traces Cholesterol Dependent Microdomain-Derived Trafficking Pathways
Source: PLoS One. 2008 Aug 13;3(8):e2933. doi: 10.1371/journal.pone.0002933 (PMC2518528; doi:10.1371/journal.pone.0002933)

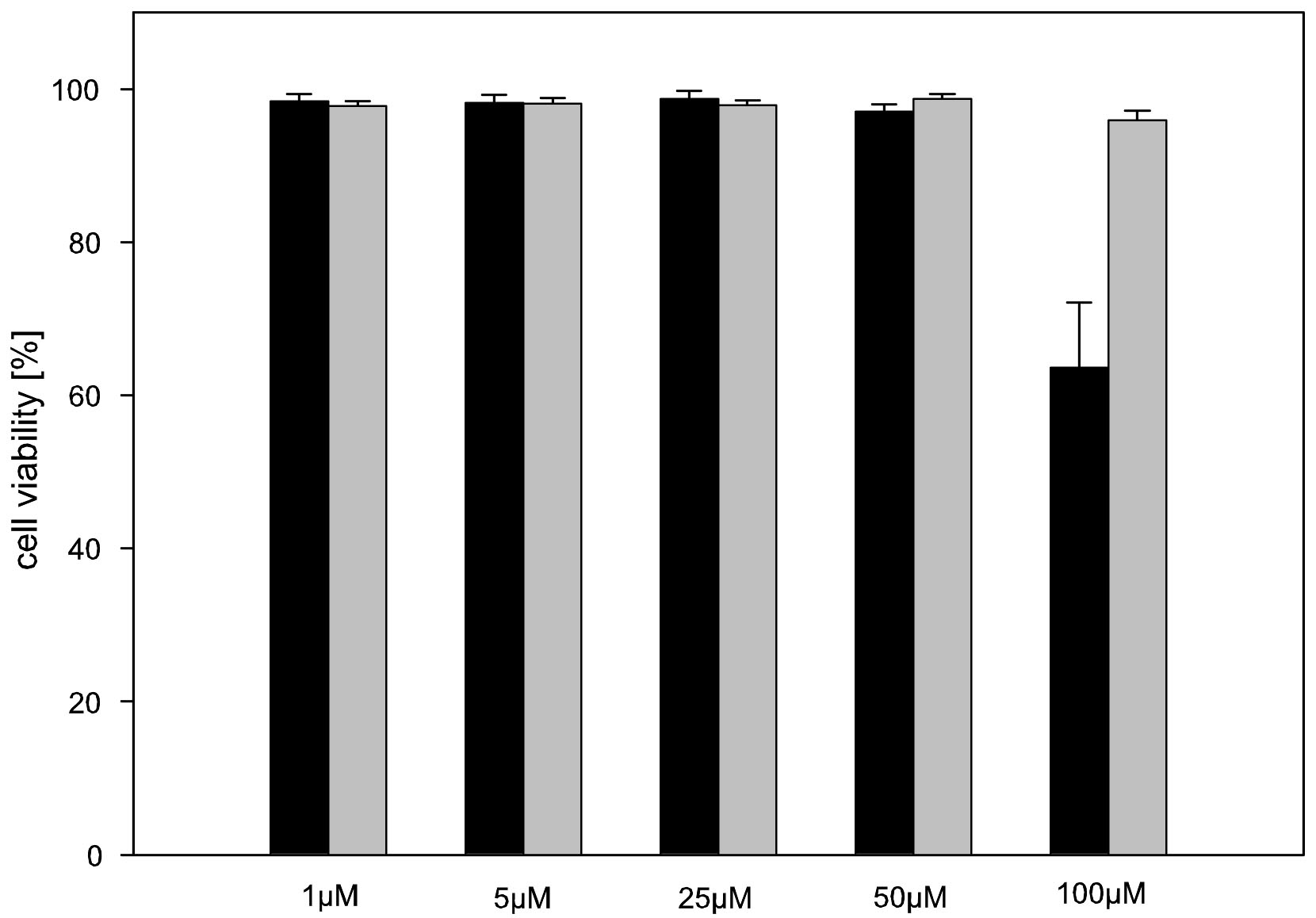

Supplement: Figure S1 — SBD treatment of cells does not affect cell viability at working concentrations (2–10 µM). Percentages of Drosophila c6 neurons negative for Sytox Green (Invitrogen) are shown for cells labeled with SBD (black bars) or SBD* (gray bars) at the indicated concentrations. (0.21 MB DOC) [file pone.0002933.s001.tif]

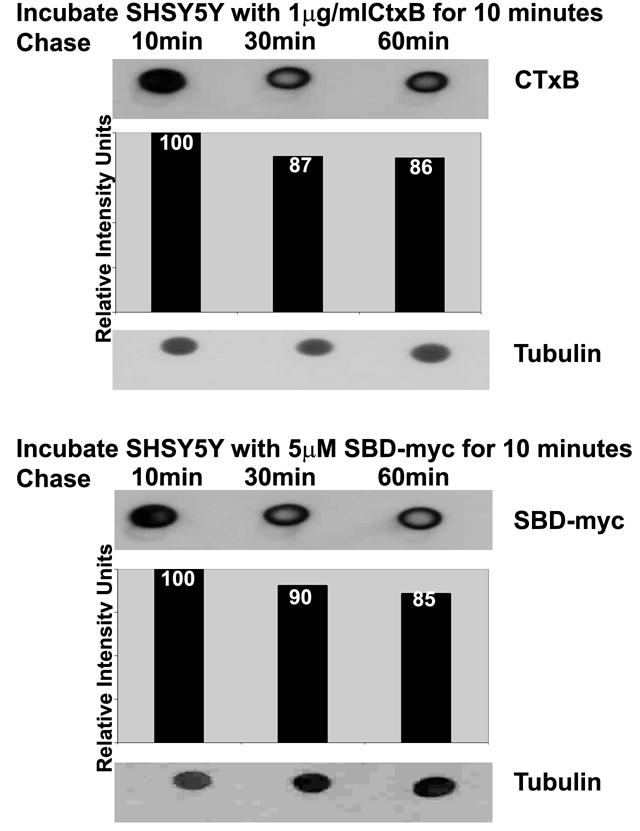

Supplement: Figure S2 — SBD is detectable by dot blots after 60 minutes of chase after uptake. SBD-myc levels in cell lysates of SH-SY5Y neuroblastoma after 10, 30, and 60 minutes of post-incubation chase are comparable on dot blots to levels of CtxB-peroxidase after similar labelling and chase in the same cell line. Quantification of myc intensity levels (see graph) indicates that 85% of SBD-myc is detectable after 60 minutes chase. Intensity levels are normalized to tht after 10 minutes chase. 86% of CtxB is detectable 60 minutes post-incubation. Detection of myc immunoreactivity indicates that internalized SBD-myc has not been degraded. Dot blots were also exposed to anti-β-tubulin as a loading control to ensure that protein amounts were comparable in the various sets. Intensity of dots was quantified using Quantity One software and represented in bar graphs. (3.21 MB TIF) [file pone.0002933.s002.tif]

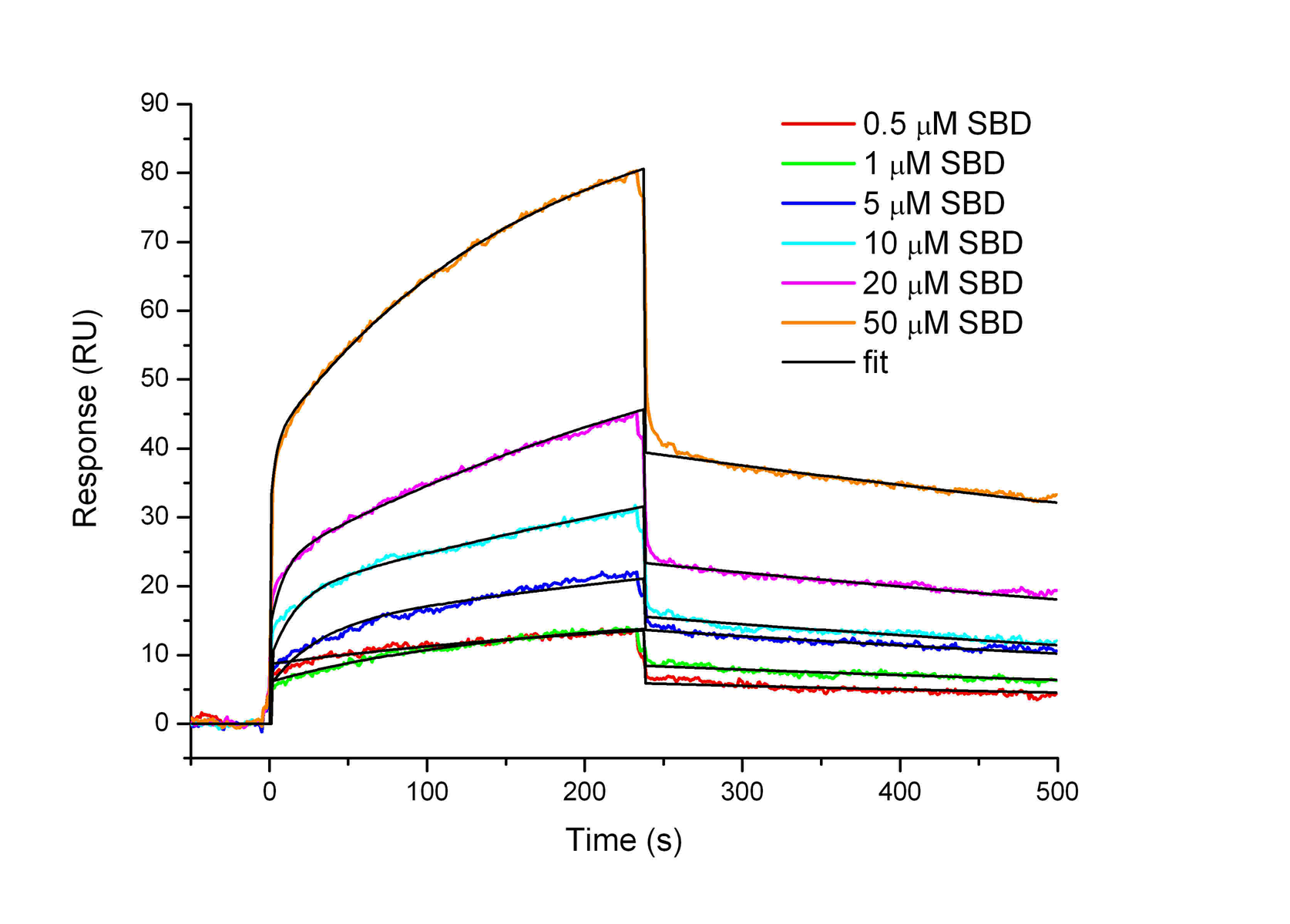

Supplement: Figure S3 — Titration curves used to calculate the binding affinity (KD) of SBD to raft-like membranes. Response signals of SBD peptide injected at various concentrations between 0.5 µM and 50 µM over a surface homogeneously covered with POPC/SM/Chol+10% GD1a liposomes. Best fit was obtained with a heterogeneous ligand model (black curves). The bulk refractive index (RI) was calculated separately contributing different bulk shift effects at the beginning and end of the injection. (6.03 MB TIF) [file pone.0002933.s003.tif]

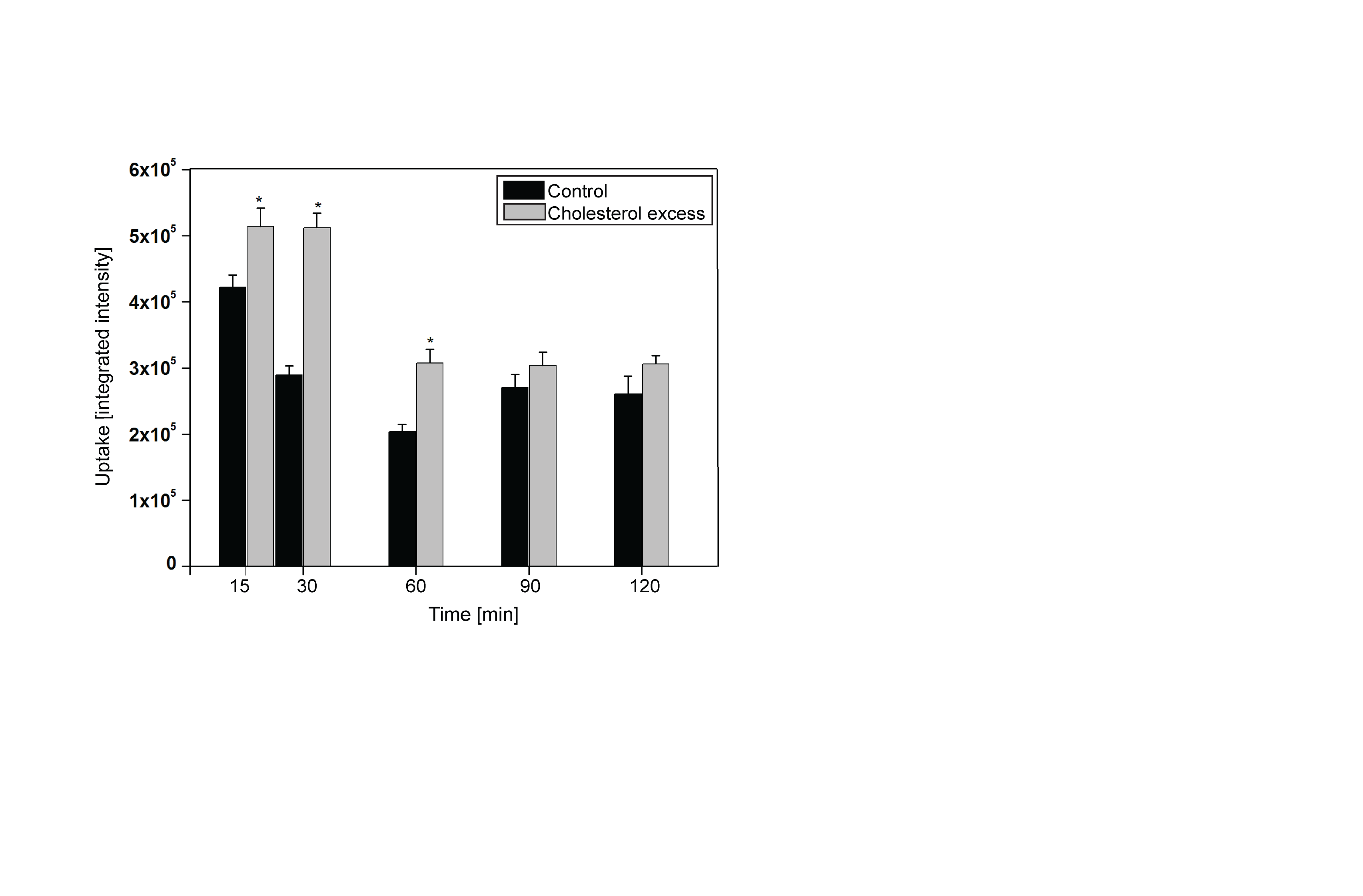

Supplement: Figure S6 — Uptake of SBD-TMR (incubated at 10 µM, 15 min, 25°C) after cholesterol overloading of c6 cells with MβCD-cholesterol complexes, assessed by measurement of total intracellular fluorescence intensity in 25 cells per time point up to 2 h after labeling, and expressed as average fluorescence intensity per pixel (quantitation was done as in Cheng et al (Cheng et al., 2006). Uptake is slightly increased in cholesterol overloaded cells (gray bars) over control cells (black bars). (0.60 MB DOC) [file pone.0002933.s006.tif]
